# Monitoring Architectural Heritage by Wireless Sensors Networks: San Gimignano — A Case Study

**DOI:** 10.3390/s140100770

**Published:** 2014-01-03

**Authors:** Alessandro Mecocci, Andrea Abrardo

**Affiliations:** Department of Information Engineering and Mathematical Sciences, University of Siena, Via Roma 56, Siena 53100, Italy; E-Mail: abrardo@dii.unisi.it

**Keywords:** cultural heritage and architectural buildings, real-time monitoring, wireless sensor networks, distributed data processing, mac protocols, remote control

## Abstract

This paper describes a wireless sensor network (WSN) used to monitor the health state of architectural heritage in real-time. The WSN has been deployed and tested on the “Rognosa” tower in the medieval village of San Gimignano, Tuscany, Italy. This technology, being non-invasive, mimetic, and long lasting, is particularly well suited for long term monitoring and on-line diagnosis of the conservation state of heritage buildings. The proposed monitoring system comprises radio-equipped nodes linked to suitable sensors capable of monitoring crucial parameters like: temperature, humidity, masonry cracks, pouring rain, and visual light. The access to data is granted by a user interface for remote control. The WSN can autonomously send remote alarms when predefined thresholds are reached.

## 1. Introduction

A WSN comprises a set of “intelligent” nodes each equipped with a radio transceiver for wireless communication, an ultra low power micro-controller for data preprocessing, and a flash memory to store data. The nodes automatically connect each other through wireless links so enabling distributed data processing and remote data transmission. This is why during the last decade the interest about WSNs has been witnessed both by industry and academic research [1]. The nodes are self powered, small, and light, do not need sophisticated network infrastructures and use flexible network protocols. This is why they constitute ideal building blocks in those situations where fast deployment, mimicry, and real-time data gathering, are important prerequisites. This is why WSNs have been proposed for a variety of interactive communication and sensing applications in many real-world scenarios (e.g., [2–4]).

Cultural Heritage and Architectural Buildings, represent a natural and important application field for WSNs, due to the need of gathering distributed data through an almost invisible infrastructure that should be easy to deploy and capable of giving automatic alarms in case of possible danger.

To fully exploit such capabilities, however, a lot of challenging tasks need to be solved. The power supply is one of the problems. Usually, standard alkaline batteries are used inside each node and their replacement may be not trivial, in particular when large and tall buildings are involved. Both the hardware and software must be accurately designed to extend the lifetime of each node and hence the operation of the overall network. Moreover, the remote user should be capable to program and schedule the tasks of each node according to the environmental changes.

**Figure 1.**
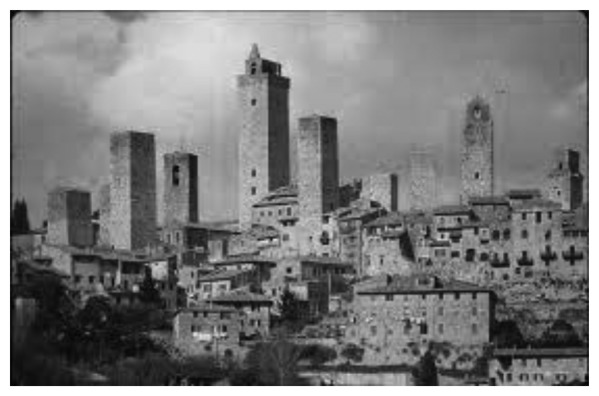
San Gimignano and its towers.

In this paper we describe an architecture for gathering real-time data about the structural health of buildings relevant from a Cultural Heritage point of view. In particular, we have focused our attention on the medieval village of San Gimignano, very well known worldwide for the incredible number of ancient towers (Figure 1).

We have developed a WSN-based system that grants the acquisition of accurate “building-health” parameters in real-time. Specifically, we selected five crucial parameters that give information about the heritage-buildings conservation state, *i.e.*, temperature, humidity, visual light, masonry cracks, and pouring rain.

## 2. San Gimignano—The “Rognosa” Tower

**Figure 2.**
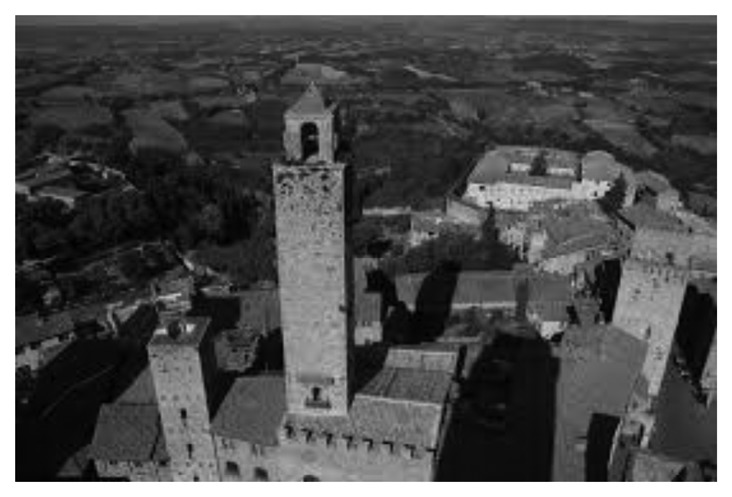
San Gimignano, the “Rognosa” tower.

San Gimignano is a medieval village nearby Siena in Tuscany, Italy, inscribed on the UNESCO list of World Heritage Sites. It is known worldwide for the architectural heritage and in particular, for its medieval towers. This important heritage needs constant monitoring. Prompted by the suggestion of experts in the field of structural conservation, the proposed system has been developed to analyze the impact of atmospheric conditions on building health. After laboratory tests, the system has been initially installed on the east fortified bastion of San Gimignano and then moved to the so called “Rognosa” tower (see Figure 2), in Piazza Duomo (Duomo Square), during the Summer of 2010.

## 3. Hardware Architecture

**Figure 3.**
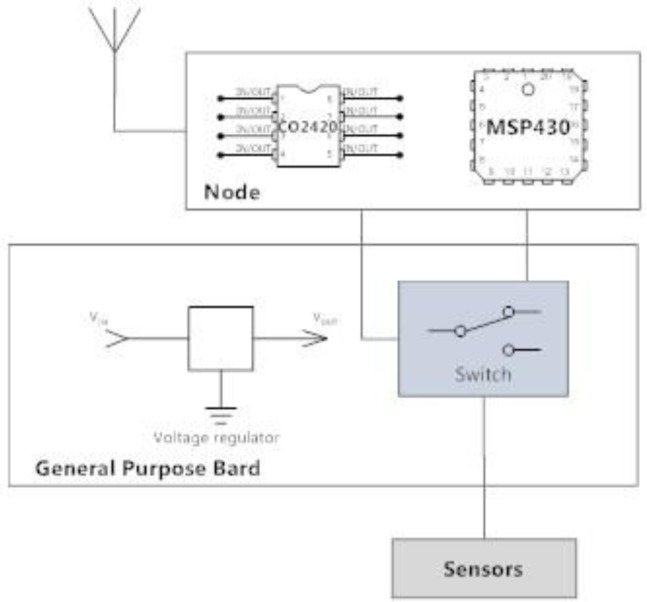
Schematic diagram of a WSN node with sensors.

The actual WSN is based on nodes able to exchange data via ChipCon CC2420 digital radio. Each node is equipped with an MSP430 micro-controller [5] and is interfaced to a sensor platform by means of a general purpose board and a 40 pins Molex (see Figure 3). This architecture allows us to connect different types of sensors to each SMARTBRICK. In particular, we can simultaneously connect analog and digital sensors (GIO, I2C, SPI).

The board regulates both the node and sensor voltage, while an electronic switch, controlled via a dedicated digital line (GIO), is used to shut down the sensors when unused. In this way it is possible to save power and dramatically extend the operation life of the whole WSN. The voltage regulator gives 3.3 V in output, which is the optimal voltage for the nodes and the sensors. In fact, the response of the sensors strictly depends on the input voltage that must be accurately stabilized.

An IP67 box (120 × 40 × 20 mm), houses the whole node; such a box has been named SMARTBRICK. A 6 V voltage supply has been selected as a compromise between the battery lifetime and the power consumption at the regulator. In fact, as the voltage level increases the lifetime increases (it takes longer before the voltage drops to the limiting level for the sensors, *i.e.*, 3.3 V in our case), but at the same time the power dissipated by the regulator increases so decreasing the battery life. In the actual implementation the system power consumption is constantly monitored by measuring the voltage level of all batteries. This information is used in order to appropriately decide the policy used to reconfigure the nodes of the network depending on the effective battery level of each of them.

The possibility of interconnecting different sensors through the general purpose board, grants the option of gathering data in a very flexible and efficient way, depending on the actual monitoring task and on the conformation of the architectural building to be controlled. In particular, we have defined three different kinds of SMARTBRICKs.

## 4. Masonry Crack SMARTBRICK (CSB)

**Figure 4.**
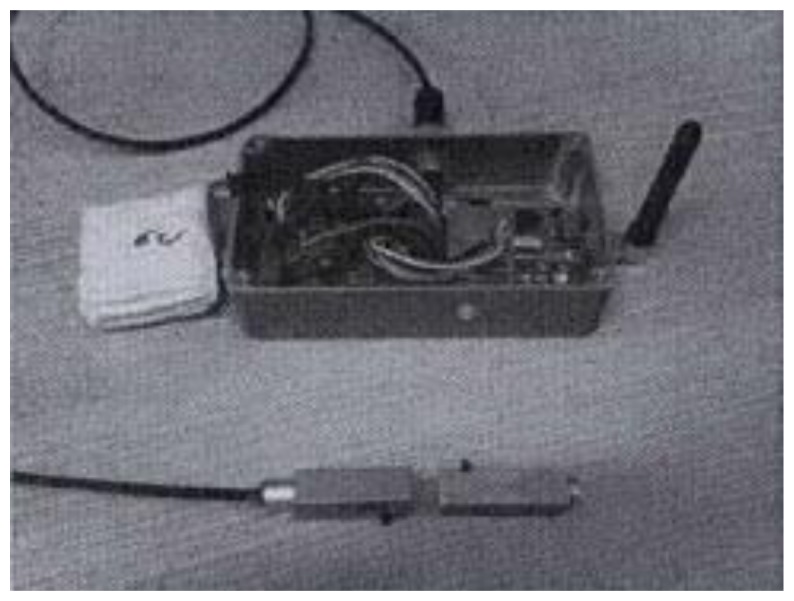
WSN node with a masonry crack sensor.

Masonry cracks are important indicators of the health state of a building and should be measured with an accuracy of about 1/10th millimeter. Such level of accuracy has been obtained by means of a Hall effect analog sensor coupled with a magnet (see Figure 4). The ADC converter and the magnet are the main roots of this resolution limit. The magnet has been selected to guarantee a working range (from 0.3 up to 2 cm) and high stability. The Hall probe is small (1 cm × 7 cm), cheap (about 2$), and has a low power consumption (about 4mA of draining current). Moreover, no warm-up is needed so real-time response is granted. To correct for the dependence on environmental conditions, the crack measurement system is equipped with a digital low power temperature sensor and a sensor for relative humidity.

## 5. Rain Gauge SMARTBRICK (RSB)

**Figure 5.**
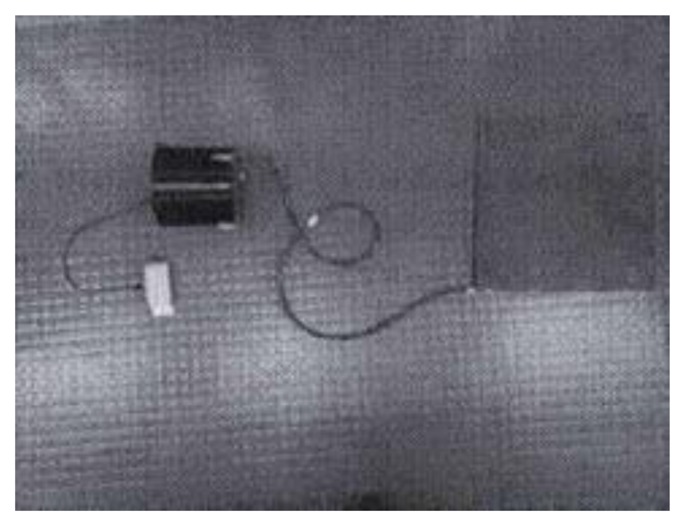
WSN node with a rain gauge sensor.

Long term damage can be given by heavy rain impacting the building surface, particularly on old buildings. A digital rain gauge insisting on a 50 cm × 50 cm planar surface that uses a drainpipe to collect water, has been developed (see Figure 5). In the absence of rain, the sensor consumes negligible energy, while during rain it drains approximately 2 mA. Every 10 mL of rain, a digital interrupt is notified to the node.

## 6. Light Irradiation SMARTBRICK (LSB)

**Figure 6.**
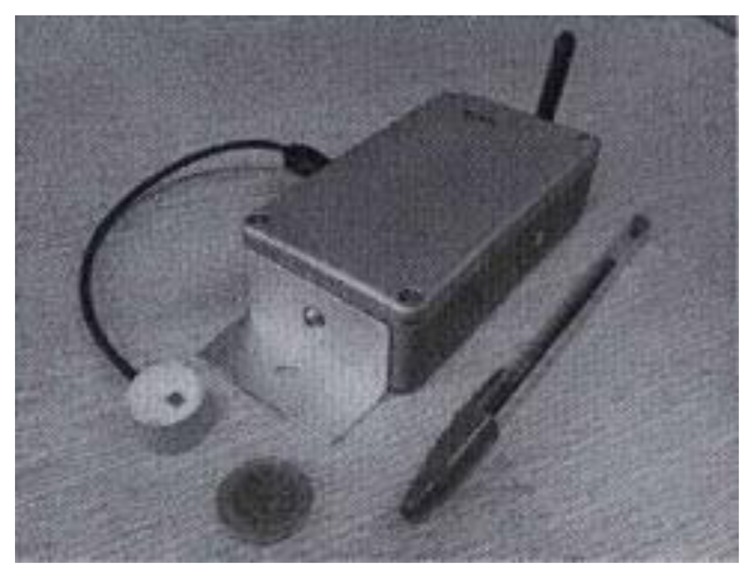
WSN node with a light sensor.

Some special surfaces (e.g., frescoes) are damaged by light beams particularly in the long-term time scale. To account for this a low power digital light sensor has been developed that drains about 0.3 mA current (see Figure 6). Like the CSB module, each LSB is coupled with a low power, temperature, and relative humidity sensor.

A special attention has been devoted to the minimization of power consumption. The measured performance is 22 mA when the radio is active, 4 mA when sensing, and 28 µA when sleeping.

## 7. Software Functionalities

The control software has been developed by using TinyOS [6]. This OS is easy to use, it is well supported, and very reliable [7]. From a telecommunication point of view it provides an IEEE 802.15.4 like stack [8] that defines the MAC and PHY layers in WSNs. Under TinyOS we developed a configuration manager inside each SMARTBRICK, a configuration manager into the base station (BS), the drivers for each sensor (temperature, humidity, masonry cracks, light) and the web interface to remotely control each SMARTBRICK and access the data.

**Figure 7.**
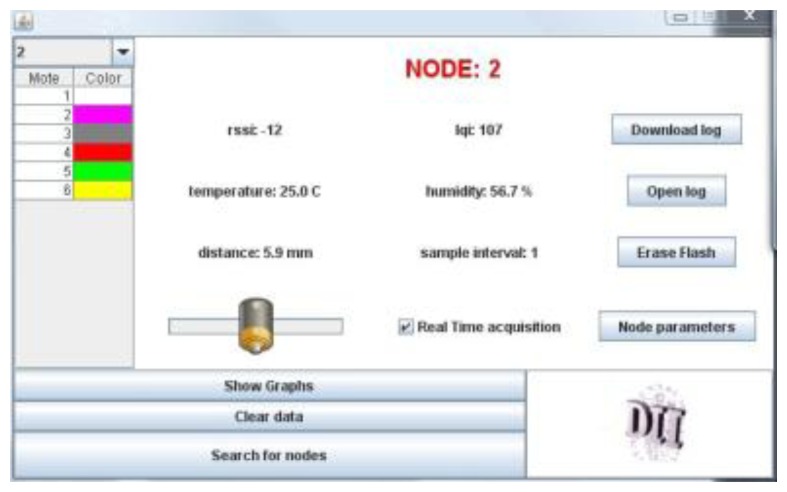
Java based interface for the WSN.

**Figure 8.**
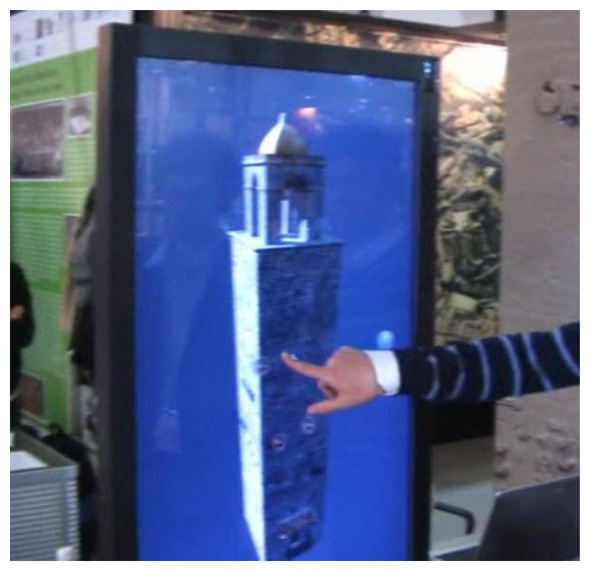
3D interactive interface based on touch screen.

In our system the BS acts as a router between the IEEE 802.15.4 network, and the UMTS/GPRS network. Because the BS acts as a server, the Java programming language has been used. As a web server the BS can transmit data to clients over the internet through the UMTS/GPRS connection. By means of a predetermined set of commands, the web interface sets the sampling data rate for each kind of sensor and the data collection rate; these rates can be modified in real–time and can be different SMARTBRICK by SMARTBRICK. The remote controller, has been implemented both as a Java applet (currently as a web client in a web page, see Figure 7), or as a user-friendly 3D interface (touch screen based, Figure 8), that grants a full interactive remote control over the network. In particular, the 3D interface uses a three-dimensional model of the “Rogonosa” tower animated by the real-time data coming from the WSN so reproducing, instant by instant, the current state of the tower itself. This is another interesting extension of the 3D representation metaphor that we have developed, namely: a 3D virtual representation that *lives the life of the actual object*. In fact, the illumination of the 3D interface changes according to the actual light intensity gathered through the WSN sensors, the surface reflectance changes according to the rain level, the cracks (if you zoom the model) reflect the current state as given by the corresponding masonry crack sensor.

The battery consumption is controlled by using the Low Power Listening (LPL) protocol [9] to duty cycle the radio while ensuring reliable message delivery, without using any synchronization protocol. The LPL protocol forces the receiver node to turn on the radio for a short time during each duty cycle interval (DC). Every DC, the SMARTBRICK checks the channel waiting for an incoming message. Obviously if the BS (usually main powered) wants to send a packet to a certain node, it must transmit continuously until the SMARTBRICK turns on the radio. Similarly if a node sends messages to other nodes (e.g., when multi-hop is needed), it must continuously transmit. The “always connected network state” is granted through the Collection Tree Protocol (CTP) a self-configuring multi-hop protocol able to transmit data from all nodes towards the BS [10,11]. During duty-cycling, the micro-controller goes into low power mode (LPM, 28 μ A current drain) and only the SMARTBRICK timer is active. The wake-up latency of this strategy is negligible in our application scenario.

The sensor activation is determined by a configurable sample interval (SI), with SI > > DC. The actual application software acquires data from each sensor and stores them in the internal memory for future download. The user can asynchronously request a real time data transmission, or download all the past data. In this second case, the cache memory is cleared.

The link range of each SMARTBRICK is about 30 m, which is well compatible with the building monitoring scenario.

## 8. Experimental Results

**Figure 9.**
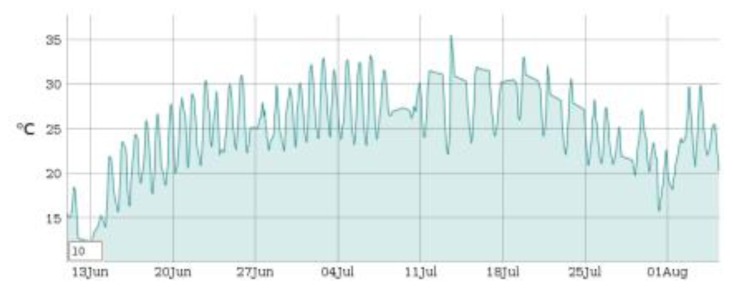
Temperature diagram: 55 days log.

**Figure 10.**
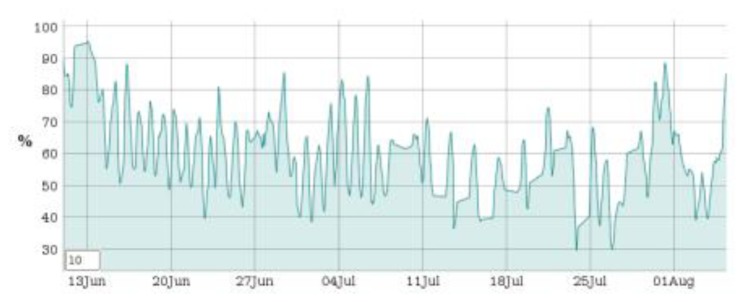
Humidity diagram: 55 days log.

**Figure 11.**
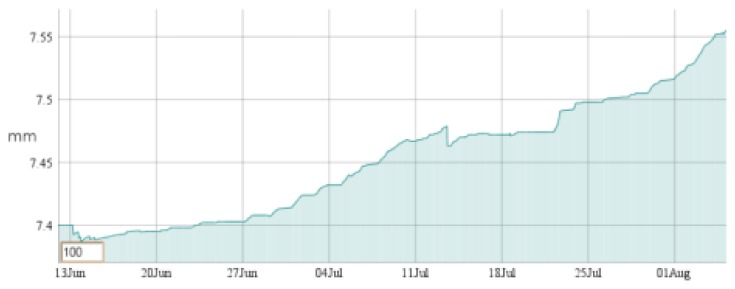
Masonry crack: 55 days log.

**Figure 12.**
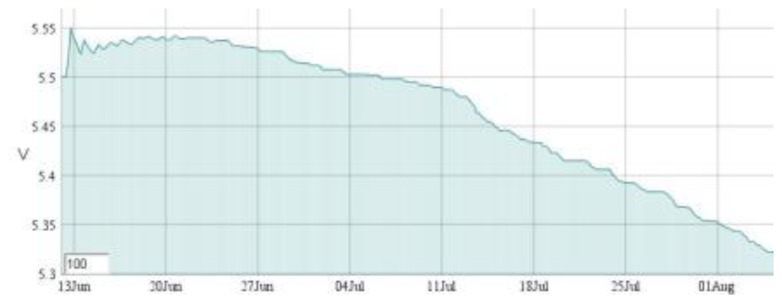
Voltage drop: 55 days log.

Six SMARTBRICKs equipped with temperature sensors and humidity sensors, three SMARTBRICKs equipped with masonry crack sensors, and one equipped with a visual light sensor and a rain sensor, have been deployed on the “Rognosa” tower in San Gimignano. Data have been gathered from the sensors every 10 min. The first diagram reports the temperature (Figure 9), the second one reports the humidity (Figure 10), and the third reports the masonry crack logs (Figure 11); all obtained during 55 days of continuous monitoring. Figure 11 shows a crack width increase of about 0.15 mm during this period. In the diagram of Figure 12 it is reported the voltage level evolution for the crack sensor. The voltage decreased about 0.25 V and, since the circuitries require a minimum of 3.3 V, we reasonably expect about one year and a half lifetime for the whole network.

## 9. Conclusions

This paper describes a WSN-based system to monitor the health state of heritage-buildings in real-time. The system has been deployed in the medieval village of San Gimignano and consists of different nodes able to measure five crucial parameters, namely: temperature, humidity, visual light, masonry cracks, pouring rain. WSNs offer a noninvasive, mimetic, and easy-to­deploy tool, particularly well-suited to monitor different parameters of interest in harsh environments. The experiments showed that an appropriate battery saving policy and the possibility to monitor and interact with the deployed network at every instant, are the principal aspects to take full advantage of such a technology. In the future particular attention will be devoted to the development of specific algoritms dedicated to the analysis of local and global trends in the signals coming from the network nodes, to forecast eventual future structural problems at a very early stage.
